# Retained Foreign Body Causing a Liver Abscess

**DOI:** 10.1155/2019/4259646

**Published:** 2019-12-14

**Authors:** Guek Gwee Sim, Sujata Kirtikant Sheth

**Affiliations:** Changi General Hospital, Singapore

## Abstract

**Introduction:**

A liver abscess caused by fishbone ingestion is extremely rare in the Emergency Department.

**Case Report:**

We report a case of a middle-aged female who presented to the Emergency Department with nonspecific symptoms. Computed tomography showed a liver abscess that had formed secondary to a fishbone. The patient was treated conservatively initially and subsequently with percutaneous drainage and finally with open drainage. Her condition improved and she was discharged from the hospital with the foreign body still in-situ.

**Conclusion:**

This case is one of six cases in literature where the patient has been discharged successfully from the hospital with a retained fishbone. It also demonstrates the difficulty of diagnosing a foreign body causing a liver abscess and the multiple treatment modalities used to treat a liver abscess caused by fishbone.

## 1. Case Report

A 56-year-old female presented to the Emergency Department after a fall. The patient reported that she had been having a fever for the last 2 days and there was associated dizziness which led to the fall. There was no loss of consciousness or head injury secondary to the fall. She also reported a non-productive cough, several episodes of non-bilious, non-bloody vomiting, and two episodes of diarrhea.

The patient's past medical history was that of chronic ischemic heart disease; type two diabetes mellitus, hyperlipidemia and hypertension. She did not have any known drug allergies. She denied drinking alcohol, smoking or use of any illicit drugs. Her travel history including traveling to India three months ago. On physical examination she looked diaphoretic, lethargic and in pain, with a blood pressure of 125/95 mmHg, pulse rate of 127 beats per minute, respiratory rate of 20 per minute, oxygen saturation of 97% on room air and a temperature of 39.7°C. An abdominal examination revealed that she was tender in the right lower quadrant and right upper quadrant with no rebound tenderness or guarding, and Murphy's sign was negative. Examination of the other systems did not reveal any abnormalities.

Based on the clinical history and examination the working diagnosis of pneumonia and possible acute appendicitis or diverticulitis was made. She was given intravenous normal saline fluid, intravenous antibiotics and analgesia. The electrocardiogram showed sinus tachycardia with nonspecific T wave inversion. The chest X-ray showed clear lungs fields and the heart size was normal. A renal panel, liver panel, full blood count, C-reactive protein, prolactin, and urine analysis were ordered. The patient had transaminitis, markedly raised inflammatory markers, and thrombocytopenia. The patient's lab results are shown in Table 1.

**Figure 1:**
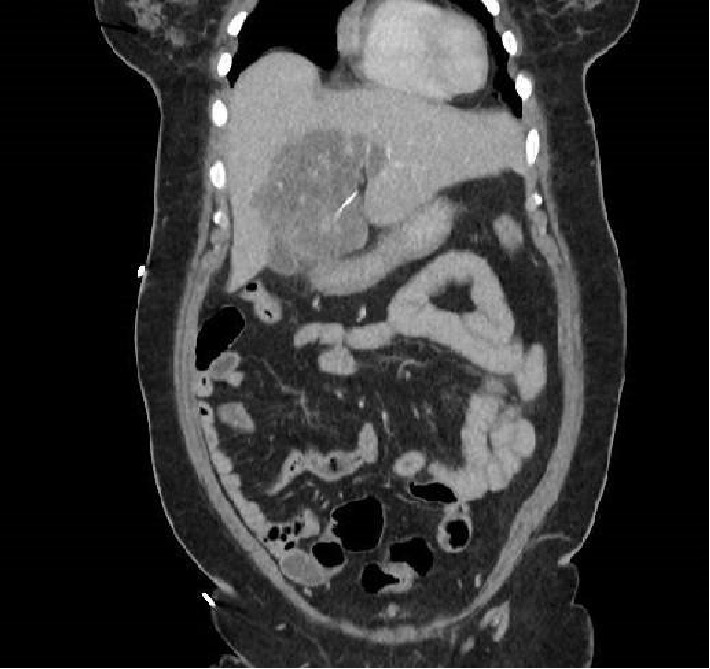
Computed tomography of the abdomen and pelvis with foreign body.

Patient was sent for a computed tomography (CT) of the abdomen and pelvis with intravenous contrast to rule out appendicitis or diverticulitis.  Figure 1 shows the CT findings for this patient. The results showed a linear radio dense foreign body within the hepatic segment, most likely a fishbone with the site of perforation possibly being the distal stomach. Surrounding the foreign body there is a well-defined hypodense region suggestive of a phlegmonous area of inflammation measuring 9.4 × 7.0 cm. No subcapsular hematoma, intra-abdominal free fluid or pneumoperitoneum was present.

The patient was then admitted to the general ward and was treated conservatively with intravenous antibiotics for 15 days. She was initially started on Ceftriaxone and Metronidazole and subsequently switched to Piperacillin/Tazobactam on Day 4 of illness. On day 10 of admission, the patient went for a repeat CT of the abdomen and pelvis and it was found that the abscess had significantly increased in size and a percutaneous catheter was inserted at this time. On day 18 the abscess was still the same size and the patient continued to spike fevers so the patient underwent open liver abscess drainage which showed a heterogeneous abscess in segment 4 extending to segment 8, but no liquid abscess was seen and no foreign body could be found. On day 27 a repeat CT of the abdomen and pelvis was done, because the patient was still spiking fevers, showing pockets of residual collection in right lobe of the liver with a foreign body still present and then a new percutaneous drain was placed. On day 29 the drain was removed because the drain output had dropped. An ultrasound was performed of the liver on day 34 and there was an ill-defined heterogenous hypoechoic area noted in the liver extending from segment 4 to 8. There was no new focal hepatic lesion. The repeat ultrasound on day 44 was done and the abscess cavity was now smaller and there was still a remnant foreign body.

The patient was successfully discharged asymptomatic on day 55. The patient remained well on follow up. She had repeat ultrasounds done at 1 month and 3 months post discharge which showed a heterogeneously hypoechoic area which had decreased in size but still had the fishbone present.

## 2. Discussion

The formation of liver abscess secondary to a foreign body ingestion is extremely rare. Most foreign bodies that are ingested pass through the gastrointestinal tract within 1 week [1]. There are a variety of foreign bodies that are ingested that have caused liver abscess. The foreign bodies ingested range from toothpicks, fishbone, needles, chicken bones, pens, and dentures. Toothpicks are the most common foreign body ingested followed by fishbones [1]. Since the first case reported by Lambert in 1898, there have been 88 cases of hepatic abscess caused by a foreign body ingestion [2]. Of these cases 33% are due to the ingestion of a fishbone [1]. As the foreign body passes through the alimentary tract there are several locations where the foreign body tends to lodge. Once swallowed, a foreign body may lodge itself in the upper aerodigestive tract, oesophagus, stomach, small bowel or colon. The most common site of impaction is usually at the level of the tonsils, although the impacted bone may be found at the base of the tongue, the vallecula or the pyriform fossa [3]. Perforations distal to the oesophagus occur in <1% of cases [4]. Once the fish bone passes through the oesophagus and pass below the level of diaphragm, the possible sites of lodgment and thus perforation include the pylorus, the duodenum, the duodenojejunal junction, the ileocecal region or any sites of congenital anomalies [5]. In this case the CT showed fat stranding between the gastric antropyloric region and the left lobe of the liver suggesting that this is the possible site of migration and penetration.

In most cases, ingested foreign bodies are asymptomatic and pass through the gastrointestinal tract without any complications within a week [6]. In Asian countries since fish is commonly consumed it is the most common foreign body that is ingested as well as the one that poses the most significant risk for gastrointestinal perforation [6]. Therefore it is difficult for the clinician to determine that this is the cause of an abscess formation. The classic signs of liver abscess fever, right upper quadrant pain and jaundice are rarely seen [7]. Instead patients present with epigastric pain, fever, chills, anorexia, nausea and vomiting or even weight loss [2]. The most common differential diagnosis before surgery is often acute appendicitis or diverticulitis [8].

There are several modalities used to diagnose foreign bodies that have penetrated from the alimentary tract. In 101 cases that were analyzed for all foreign body ingestions that have caused liver abscess, CT was the most common modality used.  Table 2 shows the breakdown of all the modalities used [9].

There is no established imaging modality that is the gold standard but CT provides a useful way of evaluating location and complications for the foreign body [9]. In this case a CT was used to discover the foreign body since the working diagnosis on initial presentation was acute appendicitis or diverticulitis.

Once the diagnosis is made there are various forms of treatment depending on the size of the abscess. Conservative management with antimicrobial agents may be attempted for abscesses less than 5 cm in size. If the liver abscess is greater than or equal to 5 cm, percutaneous drainage is recommended [3]. The bacteriological presentations of foreign-body- and nonforeign body-related pyogenic liver abscesses are different. Nonforeign body liver abscess in the majority of cases are caused by Klebsiella pneumonia and *Escherichia coli*. In foreign body related abscesses, pathogens include normal oral flora, with Streptococcus being the most common [12]. In this patient, both conservative as well as percutaneous treatments had failed, and this was followed by a laparotomy for open drainage. This resulted in improvement of the symptoms and reduction of the abscess size even though no foreign body could be retrieved. In our literature search of 54 cases of liver abscess caused by fishbones the procedures used for removal are listed in Table 3. To the best of our knowledge this is the fourth case of a liver abscess due to a foreign body ingestion that has successfully been treated without the removal of the foreign body [10, 11]. A table of all the cases of liver abscess caused by a fishbone foreign body are listed in Table 4.

## 3. Conclusion

This case is one of six cases in literature where the patient has been discharged successfully from the hospital with a retained fishbone. It demonstrates the difficulty of diagnosing an ingested foreign body as the cause of a liver abscess and the various modalities used to treat liver abscess caused by foreign bodies.

## Figures and Tables

**Table 1:** Lab values for the patient.

Renal panel (normal range)	Liver panel (normal range)	Full blood count (normal range)	Others (normal range)
Urea 6.4 (2.8–7.7 mmol/L)	Alkaline phosphate 116 (32–103 U/L)	WBC 9.6 (4.0–10.0 × 10^3^/uL)	CRP 308.8 (<3.0 mg/L)
Sodium 122 (135–145 mmol/L)	Alanine transaminase 631 (10–55 U/L)	Platelet 94 (150–450 × 10^3^/uL)	Procalcitonin 13.39 (0.00–0.50 ug/L)
Potassium 3.4 (3.5–5.3 mmol/L)	Aspartate transaminase 1224 (10–45 U/L)	Hemoglobin 10.7 (11.5–15.0 g/dL)	Urine WBC 2, RBC 12, esterase negative (WBC 0–6 cells/hpf, RBC 0–3 cells/hpf)
Chloride 80 (96–108 mmol/L)	Total bilirubin 26.3(5.0–30.0 U/L)	Hct 31 (36.0–46.0%)	
Bicarbonate 12 (19–31 mmol/L)	Albumin 31 (37–51 g/L)	Neutrophil 8.4 (2.0–7.5 × 10^3^/uL)	
Glucose 17.1 (3.1–7.8 mmol/L)		Lymphocyte 0.6 (1.0–3.0 × 10^3^/uL)	
Creatinine 107 (50–90 umol/L)			

**Table 2:** Diagnostic tools used for imaging foreign bodies.

Diagnostic tools	*n *(%)
Computed tomography	66 (65.35)
Ultrasonography	21 (20.79)
Radiographs	14 (13.86)
Laparotomy	13 (12.87)
Autopsy	7 (6.93)
Esophagogastroduodenoscopy	4 (3.96)
Colonoscopy	3 (2.97)
Endoscopic ultrasonography	1 (0.99)

**Table 3:** Procedures used to remove fishbones (reference from Table 4).

Procedure used to removed foreign body in 56 patients	*N* (%)
Laparotomy	20 (37.04)
Percutaneous abscess drainage + other procedures	16 (29.63)
Laparoscopy + other procedures	11 (20.37)
IV antibiotics	3 (5.56)
Autopsy	2 (3.70)
Endoscopy + other procedures	2 (3.70)
Colonoscopy	1(1.85)
Unknown	1 (1.85)

**Table 4:** Cases of fishbones causing liver abscess's and particulars on each case.

First Author	Year	Treatment	Symptoms	Suffering period	Size	Penetration	Bacteria	Mortality	Retained FB
Venkatesh [13]	2015	Laparotomy	Fever, abdominal pain RUQ tenderness	5 days	1.4 cm	Stomach, left liver lobe	*Klebsiella*, *Proteus vulgaris, Citrobacter freundii*, and *Alpha hemolytic Streptococcus*	No	No
Masoodi [14]	2012	Laparotomy	Fever, abdominal pain RUQ tenderness	10 days	2.5 cm	Duodenum, right liver lobe		No	No
Horii [15]	1999	Percutaneous abscess drainage and endoscopic forceps	Fever, vomiting	2 week	2.8 cm		*Streptococcus constellatus*	No	No
De la Vega [16]	2001	Autopsy	Abdominal pain, vomiting		2.5 cm	Right liver lobe		Yes	No
Tomimori [17]	2004	Laparotomy	Epigastric pain	4 weeks	1 cm	Stomach, left liver lobe	*Streptococcus constellatus*	No	No
Kessler [18]	2001	Laparotomy	Abdominal pain, vomiting	4 weeks		Duodenum, left liver lobe	*Eikenella corrodens*	No	No
Theodoropoulou [19]	2002	Endoscopy, laparoscopy, laparotomy							
Chan [20]	1999	Laparotomy	Abdominal pain			Stomach		No	No
Tsai [21]	1999	Lapartomy	Abominal pain, fever		3.7 cm	Stomach, left liver lobe		No	No
Shuldais [22]	1992					stomach		No	No
Masunaga [23]	1991	Percutaneous abscess drainage, parcial gastrectomy and lateral segmentectomy	Abdominal pain, fever, vomiting	1 week	4 cm	Stomach, left liver lobe		No	No
Gonzalez [24]	1998	Laparotomy	Abdominal pain, fever, jaundice, nausea	1 month		Stomach, left liver lobe		No	No
Aron [25]	1966	Laparotomy	Astenia, fever, jaundice	3 month	2.2 cm	Stomach, right liver lobe	*E. coli*, Proteus	No	No
Tsuboi [26]	1981	Laparotomy	Epigastic pain, weight loss	1 month	4.5 cm	Stomach, left liver lobe		No	No
Dugger [27]	1990	Autopsy	Fever, right upper abdominal pain	3 week	3 cm	Stomach, right liver lobe	*E. coli*, Proteus	Yes	No
Lee [28]	2005	Laparotomy	Epigastric pain	5 days	3.5 cm	Stomach, left liver lobe	*Streptococcus milleri*	No	No
Goh [29]	2005	Laparotomy	Fever	5 days	3 cm	Duodenum, left liver lobe	*Streptococcus milleri*	No	No
Chen [30]	2011					Duodenum			
Yang [31]	2005	IV antibiotics	Chills, fever	1 week		Left liver lobe	*Klebsiella pneumoniae* and aerobic, gram-positive *bacilli*	No	Yes
Peixoto [32]	2016	IV antibiotics	Fever, chills, RUQ pain	2 days	3.0 cm	Pylorus		No	Yes
Fan [33]	2002	Laparotomy	Fever, cough, abdominal pain	1 week	3.5 cm	Antrum, left liver lobe	*Streptococcus milleri*	No	No
Chen [12]	2013	Percutaneous abscess drainage and laparotomy	Abdominal pain, chills, fever	4 days	5.0 cm	Duodenum, left lobe liver	Strep viridans	No	No
Santos [2]	2007	Percutaneos abscess drainage, laparotomy	Abdominal pain, fever, asthenia	6 week		Antrum, left liver lobe		No	No
Clarencon [34]	2008	Percutaneos abscess drainage, IV antibiotics, laparotomy, hepatotomy	Abdominal pain	4 weeks	2.3 cm	Duodenum, liver	*Streptococcus* sp.	No	No
Ng C T [11]	2011	IV antibiotics	Acute MI	2 days				No	Yes
Chikwendu [35]	2015	Laparotomy	Abdominal pain	3 weeks	6 cm	Stomach, left liver lobe		No	No
Panebianco [1]	2015	Explorative laparoscopy	Epigastric pain, fever	2 weeks	4 cm	Antrum, left liver lobe		No	No
Kadowaki [36]	2007	Laparotomy	Fever, upper abdominal pain	1 week	2.8 cm	Hepatoduode nal fistula, left liver lobe	*E. coli* and anerobic gram positive *cocci*	No	No
Gigirey [37]	2012	Percutaneous abscess with fisbone migration to gastric lumen removal by stool	Abdominal pian RUQ pain, fever	15 days	2.5 cm	Antrum, left liver lobe		No	No
Laterre [8]	2014	Laproscopy, laparotomy	Dyspnea, fever	3 days	3 cm	duodenum	*Streptococcus hemolyticus* (group G) and *Streptococcus sanguis*	No	No
Jimenes- Fuertes [38]	2016	Laparotomy	Epigastric and RUQ pain	2 days		Duodenum, liver		No	No
Chun [32]	2016	Percutaneous abscess with laproscopy	RUQ pain, fever, chills, rigors	2 weeks		Left liver lobe		No	No
Morelli [33]	2015	IV antibiotics and laproscopy	Abdominal pain, fever, chills, jaundice		3 cm	Antrum, left liver lobe	*Streptococcus constellatus*	No	No
Akimori [34]	2013	IV antibiotics and laproscopy	Fever, malaise			Lesser curveture, liver		No	No
Kosar [42]	2014	Laproscopy and percutaneous drainage	Fever			Lesser curveture, left liver lobe			
Wu [43]	2016	IV antibiotics, laproscopy, laparotomy	Abdominal pain, fever, epigastric, RUQ pain	6 days		Duodenum, liver		No	No
Yen [44]	2010	Laparotomy	Upper abdominal pain, fever	2 weeks		Left liver lobe		No	No
Bandeira-de- Mello [45]	2018	Percutaneous abscess drainage and laproscopy	Epigastric pain, fever	6 days	2.5 cm	Lesser curveture, left liver lobe	Group C beta-*hemolytic streptococcus*, *streptococcus* sp. (viridans)	No	No
Matrella [6]	2014	Laparotomy	Epigastric pain and fever	10 days					
Motallebzadeh [46]	2014	Endoscopy and biopsy forceps	Right upper quadrant pain			Antrum, liver		No	No
Liang [47]	2011	Percutaneous drain and Laparotomy	Upper quadrant pain	1 month		Stomach, left liver lobe		No	No
Kim [48]	2010	Colonoscopy	Right upper and lower quadrant pain	2 weeks	Two 1.5 cm	Acensding colon and right liver lobe		No	No
Yu [49]	2018	Laproscopy, laparotomy	Epigastric pain, fever, anorexia, nausea and vomiting	8 days	3	Left liver lobe		No	No
Beckers [50]	2019	Percutaneous drainage and laparoscopy	Fever, right upper quadrant pain, anorexia, shivering and confusion	3 days	3.5	Left liver lobe	*Streptococcus anginosus* and *Streptococcus constellatus*	No	No
Sun [51]	2018	Laparotomy	Fever and right upper quadrant pain	3 days	3	Left liver lobe	*Streptococcus anginosusStreptococcus constellatus* and *Peptostreptococcus asaccharolyticus*	No	No
Queiroz [52]	2019	Laparotomy	Epigastric pain and fever	10 days		Gastric antrum and left liver lobe		No	No
Jarry [53]	2011	Laparoscopy, laparotomy	Right upper quadrant pain, asthenia, anorexia and fever	2 weeks	3.5	Left liver lobe		No	No
Bekki [54]	2019	Laparoscopy	Fever and anorexia		2.4	Left liver lobe	*Streptococcus anginosus*	No	No
Chen [55]	2019	Laparoscopy	Epigastric pain	2 months	1.7	Left liver lobe		No	No
Dias [56]	2018	Laparotomy	Fever and abdominal pain		2.7	Left liver lobe		No	No
Burkholder [57]	2019	Percutaneous drainage	Fever, right upper quadrant pain, nausea, vomiting and diarrhoea	8 days	2.1 cm	At level of falciform fissure	Alpha hemolytic streptococcus	No	Yes
Graça [58]	2019	Laparoscopy	Shivering and fever	3 days		Left liver lobe	*Streptococcus constellatus* and *Escherichia coli*	No	No
Sivarajah [59]	2018	Percutaneous drainage and laparotomy	Epigastric pain, nausea and vomiting	3 days	3.5	Left liver lobe		No	No
Mateus [60]	2018	Percutaneous drainage and laparotomy	Abdominal pain and constipation	3 days	5	Left liver lobe	*Streptococcus constellatus*	No	No
Mateus [60]	2018	Percutaneous drainage	Weakness, chills, myalgia and cough	2 days		Left liver lobe	*Streptococcus anginosus, Streptococcus viridans, Prevotella bivia* and *Bacteroides fragilis*	No	Yes
